# Prostaglandins as PPAR**γ** Modulators in Adipogenesis

**DOI:** 10.1155/2012/527607

**Published:** 2012-12-25

**Authors:** Ko Fujimori

**Affiliations:** Laboratory of Biodefense and Regulation, Osaka University of Pharmaceutical Sciences, 4-20-1 Nasahara, Takatsuki, Osaka 569-1094, Japan

## Abstract

Adipocytes and fat cells play critical roles in the regulation of energy homeostasis. Adipogenesis (adipocyte differentiation) is regulated via a complex process including coordinated changes in hormone sensitivity and gene expression. PPAR*γ* is a ligand-dependent transcription factor and important in adipogenesis, as it enhances the expression of numerous adipogenic and lipogenic genes in adipocytes. Prostaglandins (PGs), which are lipid mediators, are associated with the regulation of PPAR*γ* function in adipocytes. Prostacyclin promotes the differentiation of adipocyte-precursor cells to adipose cells via activation of the expression of C/EBP*β* and *δ*. These proteins are important transcription factors in the activation of the early phase of adipogenesis, and they activate the expression of PPAR*γ*, which event precedes the maturation of adipocytes. PGE_2_ and PGF_2**α**_ strongly suppress the early phase of adipocyte differentiation by enhancing their own production via receptor-mediated elevation of the expression of cycloxygenase-2, and they also suppress the function of PPAR*γ*. In contrast, PGD_2_ and its non-enzymatic metabolite, Δ^12^-PGJ_2_, activate the middle-late phase of adipocyte differentiation through both DP2 receptors and PPAR*γ*. This paper focuses on potential roles of PGs as PPAR*γ* modulators in adipogenesis and regulators of obesity.

## 1. Introduction

Obesity is a major health concern worldwide [1] and is associated with the development of a number of pathological disorders such as type 2 diabetes, hypertension, and cardiovascular disease [2–4]. Excess adipose tissue can be the consequence of both an increased number (hyperplasia) and an enlarged size (hypertrophy) of adipose cells. A major role of adipocytes is to store large amounts of triglycerides during periods of energy excess and to mobilize these depots during periods of nutritional deprivation [2–4]. Moreover, adipocytes are highly specialized cells that secrete various adipocytokines, whose release largely reflects the amounts of stored triglyceride [2, 5–8].

The regulation of adipocyte differentiation (adipogenesis) is complex and this process includes alteration of the sensitivity to hormones and the expression of a number of genes in response to various stimuli including lipid mediators. Peroxisome proliferator-activated receptor (PPAR) *γ* and CCAAT/enhancer-binding proteins (C/EBPs) are the most important transcription factors involved in the activation of adipogenesis, and they induce the expression of a number of adipogenic and lipogenic genes that participate in the control of adipogenesis [9, 10].

PPARs are members of the nuclear receptor superfamily and play critical roles in the regulation of storage and catabolism of lipids [11, 12]. To date, three types of PPAR subtypes have been identified, that is, PPAR*α*, PPAR*β*/*δ*, and PPAR*γ* [11, 12]. PPARs increase the expression of a variety of genes in various cells through heterodimerization with retinoic acid receptors or retinoid X receptors in a ligand-dependent manner [12–16]. Among them, PPAR*γ* is expressed predominantly in adipose tissue and macrophages, is closely related to the regulation of lipid and glucose metabolisms, and is associated with the control of obesity and related diseases [11, 12]. Until now, many natural and synthetic ligands for PPAR*γ* have been identified [17–19]. 15-Deoxy-Δ^12,14^-prostaglandin (PG) J_2_ (15d-PGJ_2_) was the first identified endogenous ligand for PPAR*γ*, and it activates adipogenesis in cultured cells [20, 21]. Moreover, fatty acids such as lauric acid (C12:0) and petroselinic acid (C18:1) of the saturated fatty acids [22], linolenic acid (C18:3), eicosapentaenoic acid (C20:5), and docosahexaenoic acid (C22:6) of the *ω*3 (*n*-3) family [23], arachidonic acid of the *ω*6 (*n*-6) family [22, 23], and very-long chain fatty acids [24] were later identified as other endogenous PPAR*γ* ligands that activate PPAR*γ* functions. In addition, 9-hydroxy and 13-hydroxy octadecadienoic acids (HODE), the components of oxidized low-density lipoprotein (ox-LDL), were also identified as endogenous ligands for PPAR*γ* [25, 26]. However, whether these natural molecules can function as physiological ligands of PPAR*γ* in vivo remains unknown. In addition to natural ligands, many synthetic ligands have been identified. For example, thiazolidinediones (TZDs) such as Troglitazone, Rosiglitazone, Ciglitazone, and Pioglitazone are used for the treatment of type 2 diabetes mellitus; and these ligands affect insulin resistance and glucose homeostasis by activating PPAR*γ* functions [12, 18]. However, these TZDs increase hepatic toxicity and cardiovascular risk. Finally, Troglitazone was withdrawn from the market [27]. It is still unknown whether the toxicities associated with TZDs are derived from the binding with PPAR*γ*.

**Figure 1:**
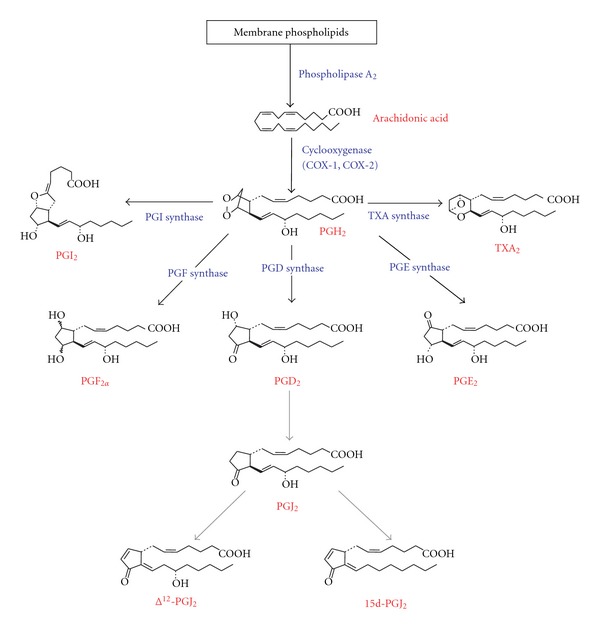
Biosynthetic pathway of prostaglandins. PGJ_2_, Δ^12^-PGJ_2_, and 15d-PGJ_2_ are converted from PGD_2_ by nonenzymatic dehydrations.

PGs are lipid mediators that play a number of physiological roles in a variety of cells. PGs are synthesized through the following three enzymatic steps (Figure 1). First, arachidonic acid is liberated from the membrane phospholipids by the action of cytosolic phospholipase A_2_ (cPLA_2_) [28]. Second, arachidonic acid is converted to PGH_2_, which is a common precursor of all prostanoids, by either cyclooxygenase- (COX-) 1 or COX-2 [29]. The activity of these enzymes is critical to determine the production rate of PGs. Third, PGH_2_ is metabolized to various PGs, that is, PGD_2_, PGE_2_, PGF_2*α*_, prostacyclin (PGI_2_), and thromboxane A_2_ (TXA_2_), by the action of specific PG synthases [29]. PGs exert a wide range of actions through their binding to specific PG receptors that belong to the G protein-coupled receptors (GPCRs) gene family [30]. GPCRs span cell membranes via seven transmembrane-spanning segments and are the most important therapeutic targets. In this decade, the functions of PGs in the regulation of adipogenesis have been extensively investigated. Elucidation of the molecular mechanisms underlying adipogenesis may provide strategies for reducing the prevalence of obesity. This paper focuses on the recent advances in our understanding of the function of PGs as modulators of PPAR*γ* in the regulation of adipogenesis.

## 2. Roles of COXs in Adipocytes

COX consists of two isozymes, COX-1 and COX-2, and is the rate-limiting enzyme in the PG biosynthesis [29]. COX-1 is constitutively expressed in most cells including adipocytes, whereas COX-2 expression is induced by various stimuli [29] and transiently activated in the early phase of adipogenesis, followed by lowered expression during adipogenesis [31]. There have been a number of reports regarding the contribution of COX isozymes to the regulation of adipocyte differentiation. However, the roles that COX-2 plays during adipogenesis are still controversial. 

In cell-based studies, Yan et al. demonstrated that inhibition of COX activities by their selective inhibitors, for example, SC-560 for COX-1, and NS-398 and Celecoxib for COX-2, enhances adipocyte differentiation via an increase in the mRNA levels of PPAR*γ* and C/EBP*α*. Thus, both COX-1 and COX-2 participate in the regulation of adipogenesis [32]. Moreover, in 3T3-L1 cells stably expressing COX-2 in the antisense direction, lipid accumulation is enhanced during adipogenesis with elevated expression of adipogenic genes such as PPAR*γ* and C/EBP*α*. In addition, this enhancement of lipid accumulation in antisense COX-2-expressing cells can be reversed by cotreatment with either antiadipogenic PGE_2_ or PGF_2*α*_ [33]. 

In contrast, when 3T3-L1 cells are pretreated before the initiation of adipocyte differentiation or treated during the clonal expansion phase with SC-58236, a selective COX-2 inhibitor, and then caused to differentiate into adipocytes, lipid accumulation is reduced along with repressed expression of the adipogenic fatty acid-binding protein 4 (FABP4, also called aP2) gene [34]. In contrast, a selective COX-1 inhibitor, SC-58560 does not have any effect on adipogenesis. Additionally, when 3T3-L1 cells are caused to differentiate into adipocyte in a medium containing each of two selective COX-1 and COX-2 inhibitors that are added after the clonal expansion phase, adipogenesis is not affected. Thus, inhibition of COX-2 activity suppresses adipocyte differentiation by repressing the clonal expansion phase [34].

In in vivo studies, overexpression of COX-2 in white adipose tissue (WAT) increases de novo recruitment of brown adipose tissue (BAT) and energy expenditure, while suppressing the high fat diet-induced gain in body weight [35]. Also, Ghoshal et al. reported that in COX-2 gene-knock-out mice, their total body weight is significantly lower than that of wild-type mice, along with reduced expression of adipogenic genes such as those of PPAR*γ* and lipoprotein lipase [36]. In addition, PGD_2_ and 15d-PGJ_2_ levels in cells prepared from adipose tissues of COX-2 gene-knock-out mice and placed in primary culture are reduced as compared with those in wild-type mice [36]. Thus, further studies are needed to elucidate the precise functions of COXs in the regulation of adipogenesis.

## 3. Repression of the Early Phase of Adipogenesis by PGF_2 **α**_

PGF_2*α*_ and PGE_2_ suppress the differentiation of adipocytes and exert their functions as antiadipogenic agents exert by acting through their specific FP [37–41] and EP4 [42, 43] receptors, respectively. 

PGF_2*α*_ is synthesized by a variety of PGF synthase (PGFS) activity-carrying enzymes [44], for example, aldoketo reductase (AKR) 1B3 [45], AKR1B7 [46], and prostamide/PGFS [47] in mice. In humans, AKR1C3 acts as a PGFS in adipocytes and is associated with the suppression of adipogenesis through inhibition of PPAR*γ* function [48]. Although PGFS has never been identified in adipocytes, we and another group identified AKR1B3 [31] and AKR1B7 [49] as being PGFSs in adipocytes. 

AKR1B3-produced PGF_2*α*_ is detected in preadipocytes and its level is enhanced with a peak at 3 h after the initiation of adipogenesis and then decreases [50], indicating that PGF_2*α*_ suppresses an early phase of adipogenesis. Fluprostenol, an FP receptor agonist, clearly reduces the expression of PPAR*γ* and its target genes [31, 50]. Moreover, this Fluprostenol-mediated suppression of the gene expression is cleared by cotreatment with AL8810, an FP receptor antagonist, indicating that PGF_2*α*_ inhibits adipocyte differentiation of 3T3-L1 cells by acting through an FP receptor.

AKR1B7 gene-knock-out mice display excessive adiposity resulting from adipocyte hyperplasia/hypertrophy and exhibit high sensitivity to diet-induced obesity. Treatment of 3T3-L1 cells or AKR1B7 gene-knock-out mice with Cloprostenol, an FP receptor agonist, decreases adipocyte size and inhibits the expression of lipogenic genes [49]. 

The precise molecular mechanism of PGF_2*α*_-mediated suppression of adipogenesis has been investigated. PGF_2*α*_ represses the function of PPAR*γ* by causing its phosphorylation via FP receptors [50]. In addition, Fluprostenol enhances the expression of COX-2 via activation of the mitogen-activated protein kinase (MEK)/extracellular signal-regulated kinase (ERK) 1/2 pathway. Moreover, promoter-luciferase and chromatin immunoprecipitation assays demonstrated that PGF_2*α*_-derived COX-2 expression is activated by the binding of cAMP-responsive element binding protein (CREB) to the promoter region of the COX-2 gene in 3T3-L1 cells [50]. Thus, the MEK/ERK-CREB cascade forms a positive feedback loop, one that probably plays a critical role in the suppression of the early phase of adipogenesis by elevating the de novo production of antiadipogenic PGF_2*α*_. 

## 4. Suppression of the Early Phase of Adipogenesis by PGE_2_

PGE_2_ is also known to suppress adipogenesis through suppression of PPAR*γ* function. PGE_2_ and an EP4 agonist, AE1-329, increase the intracellular cAMP levels in preadipocytes in a dose-dependent manner [42]. Moreover, AE1-329 decreases the expression of adipogenic genes such as PPAR*γ* and C/EBP*α* [51]. The inhibitory effect of PGE_2_, but not that of Fluprostenol, is reversed by the addition of an EP4 antagonist, AE3-208 [42], indicating that PGE_2_ suppresses adipogenesis through the EP4 receptor. Although the functions of PGE_2_ and the expression of the functions of PGE_2_ and the expression of PGESs have been investigated in adipocytes [27, 52, 53], the PGE_2_-producing enzyme in adipocytes has never been identified. To date, three major PGESs have been identified [54, 55]. Microsomal PGES-1 (mPGES-1) is a member of the membrane-associated proteins in eicosanoid and glutathione metabolism (MAPEG) protein family [56] and produces PGE_2_ in response to various stimuli [57]. Microsomal PGES-2 (mPGES-2) has also been identified and its expression is high in the heart and brain [58]. Cytosolic PGES (cPGES) is constitutively and ubiquitously expressed in various cells [59]. 

PGE_2_ production is detected in preadipocytes and increases during the early phase of adipogenesis with a peak at 3 h after the initiation of adipogenesis; and mPGES-1 is expressed in these cells, with its mRNA and protein levels being consistently detected during adipogenesis. Finally, we found that mPGES-1 is responsible for the production of PGE_2_ in adipocytes [60]. This result is consistent with results showing that treatment of mouse embryonic fibroblast (MEF) cells with PGE_2_ for the first two days of adipocyte differentiation is enough to suppress adipocyte differentiation, with reduced expression of the PPAR*γ*2 gene and reduced accumulation of intracellular lipids [43]. 

In wild-type mouse MEF cells, inhibition of endogenous PG synthesis by indomethacin enhances adipocyte differentiation, and this enhancement is reversed by the addition of PGE_2_. In MEF cells prepared from EP4 receptor gene-knock-out mice, adipocyte differentiation is elevated, and no more enhancement of adipocyte differentiation is observed following treatment with indomethacin. Thus, PGE_2_-EP4 receptor signaling suppresses the early phase of adipocyte differentiation in MEF cells [43].

## 5. Synergistic Suppression of Early Phase of Adipogenesis by PGF_2 **α**_ and PGE_2_

Both PGF_2*α*_ and PGE_2_ suppress the early phase of adipogenesis, and so we investigated the synergistic regulation of these PGs in 3T3-L1 cells. The increased production of PGF_2*α*_ and PGE_2_ in the early phase of adipogenesis is a consequence of the elevated expression of the COX-2 gene [61]. PGF_2*α*_ forms a positive feedback loop that coordinately suppresses the early phase of adipogenesis through the increased production of antiadipogenic PGF_2*α*_ and PGE_2_, both of which inhibit PPAR*γ* function. In addition, PGE_2_ also enhances the production of PGF_2*α*_ and itself through the elevation of the expression of the COX-2 gene in an EP4 receptor-mediated fashion. Moreover, when the cells are caused to differentiate into adipocytes in medium containing both PGF_2*α*_ and PGE_2_, the expression of the adipogenic genes is decreased to a greater extent than when the cells are cultured in a medium containing either of them. Thus, PGE_2_ and PGF_2*α*_ synergistically suppress the early phase of adipogenesis through a self-amplifying loop, triggered by PGF_2*α*_-FP receptor and PGE_2_-EP4 receptor couplings and activation of the COX-2 gene expression in 3T3-L1 cells [61].

However, Inazumi et al. demonstrated that the differentiation of MEF cells prepared from FP receptor gene-knock-out mice is almost the same as that in these cells from wild-type mice and still shows sensitivity to indomethacin, indicating that FP receptor-mediated suppression is not directly associated with the regulation of adipocyte differentiation in MEF cells [43]. Therefore, the regulation of suppression of adipogenesis by PGE_2_ and PGF_2*α*_ might occur in a cell-type-dependent manner.

## 6. Acceleration of Adipocyte Differentiation by PGD_2_ and Its Metabolites

PGD_2_ acts as an allergic and inflammatory mediators and is produced in a variety of cells such as mast cells, macrophages, and adipose cells [62, 63]. PGD_2_ is produced from PGH_2_ by the action of PGD synthases (PGDSs), enzymes that catalyze the isomerization of the 9,11-endoperoxide group of PGH_2_ to PGD_2_. Two distinct types of PGDSs have been identified. One is hematopoietic PGDS (H-PGDS), which is abundantly expressed in mast cells and Th2 cells [64]. The other is L-PGDS, which is detected abundantly in the brain, male genital organs, and heart [62, 63].

PGD_2_ has been considered a candidate for a molecule that acts as an endogenous inducer of adipogenesis, basically because 15d-PGJ_2_, one of its metabolites, has been identified as a ligand for PPAR*γ* and activates adipogenesis in vitro [20, 21]. PGD_2_ is nonenzymatically metabolized to PGs of the J series, that is, PGJ_2_, Δ^12^-PGJ_2_, and 15d-PGJ_2_. However, the concentrations of 15d-PGJ_2_ required for the activation of PPAR*γ* reported in most of the literature are much higher (2.5–100 *μ*mol/L) than those of conventional PGs (pmol/L range); and 15d-PGJ_2_ is present in vivo at a low level that is insufficient for activation of adipocyte differentiation [65], whose finding is consistent with our current results indicating that 15d-PGJ_2_ is not detectable in adipocytes [60]. Recently, we identified Δ^12^-PGJ_2_ as being the dominant PGD_2_ metabolite in differentiated adipocytes [60], in good agreement with recent results showing that Δ^12^-PGJ_2_ is produced in adipocytes and activates the expression of adipogenic genes in 3T3-L1 cells [66]. 

PGD_2_ is synthesized by the action of L-PGDS in adipocytes [67]. However, another PGDS, H-PGDS may not be involved in the production of PGD_2_ in adipocytes, because the expression level of H-PGDS is very low during adipogenesis. Although the function of PGD_2_ or L-PGDS in vitro has been extensively investigated, the in vivo function is still controversial. Ragolia et al. reported that adipose size is increased in L-PGDS gene-knock-out mice under normal and high-fat diet feeding. Moreover, L-PGDS gene-knock-out mice become glucose intolerant and insulinresistant. Also the serum adiponectin level is decreased in such mice [68]. Adipocytes isolated from L-PGDS gene-knock-out mice are significantly less sensitive to insulin-stimulated glucose transport. Thus, L-PGDS is an important mediator of muscle and adipose glucose transport which is modulated by glycemic conditions and plays a significant role in the glucose intolerance associated with type 2 diabetes [69]. Furthermore, Tanaka et al. showed that L-PGDS gene-knock-out mice have a significantly increased body weight when fed high-fat diet and the size of adipocytes in the subcutaneous and visceral fat tissues is significantly enlarged [70]. 

In contrast, Fujitani et al. demonstrated that transgenic mice overexpressing human H-PGDS, which produce plenty of PGD_2_ in every tissue including adipose, become obese under high-fat diet feeding but that obesity is not observed under normal diet feeding [71]. Serum leptin, insulin, and adiponectin levels are increased in these PGD_2_-overproducing mice. Moreover, their triglyceride level is decreased by about 50% as compared with that in WT mice. Moreover, the PGD_2_-overproducing mice show increased insulin sensitivity [71]. Furthermore, the epididymal adipose tissue mass of COX-2 gene-knock-out mice is decreased. PGD_2_ and the levels of PGD_2_ metabolites are also decreased in the adipose tissue of these mice. Thus, reduced adiposity in COX-2 gene-knock-out mice results from the inhibition of the production of PGD_2_ and its metabolites required for PPAR*γ* activation [36]. This discrepancy may be derived from a variety of physiological functions of PGD_2_ in the body. Therefore, the adipocyte-specific function of PGD_2_ and/or L-PGDS in the regulation of obesity should be further clarified.

## 7. Activation of Adipogenesis in Adipose-Precursor Cells by PGI_2_

PGI_2_ activates the protein kinase A (PKA) pathway by binding to its IP receptor and enhances the differentiation of adipose precursor cells [72, 73]. The activation of IP receptors upregulates the expression of C/EBP*β* and C/EBP*δ*, both of which are critical for the progression of the early phase of adipogenesis and directly activate the expression of the PPAR*γ* and C/EBP*α* genes for maturation of adipocytes [9, 10]. Moreover, IP receptor gene-knock-out mice fed a high-fat diet do not show any changes in body weight, fat mass, or adipose size [74, 75]. Therefore, PGI_2_ activates the progression of adipogenesis in the adipose precursor cells through the enhancement of the expression C/EBP*β* and C/EBP*δ* via the cAMP-PKA pathway.

## 8. Conclusion

**Figure 2:**
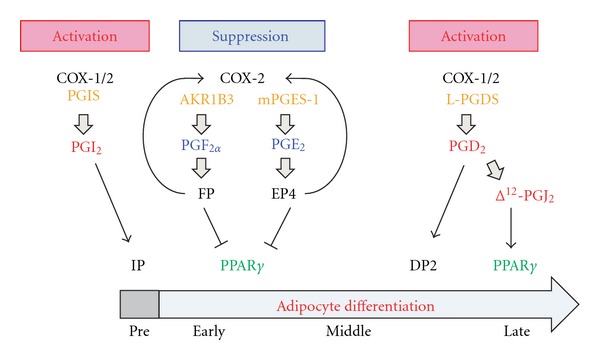
Regulation of adipogenesis by prostaglandins. *“Pre*” indicates adipocyte precursor cells. *“Early*,*”* “*Middle*,*”* and “*Late”* mean early, middle, and late phases of adipogenesis, respectively.

PGs are involved in the regulation of adipogenesis and act as modulators of PPAR*γ* functions. The regulation of adipogenesis by PGs is very complex, because PGs regulate adipogenesis both positively and negatively. In the early phase of adipogenesis, PGF_2*α*_ and PGE_2_ suppress the progression of adipogenesis, and their receptor-mediated mechanisms leading to suppressed PPAR*γ* function have been well elucidated. In contrast, PGD_2_ and its metabolites activate the middle-late phase of adipogenesis (Figure 2). In addition, recently we found that PGD_2_ and its metabolite Δ^12^-PGJ_2_ accelerate adipogenesis by acting through DP2 (CRTH2; chemoattractant receptor homologous molecule of Th2 cells) receptors and PPAR*γ*, thus, indicating that when elucidating the function of a given PG, the roles of not only it but also those of its metabolites should be considered.

All PGs function through their specific G protein-coupled receptors and PPAR*γ*. Although their receptor agonists and antagonists are functional in the cultured adipocytes (in vitro), in vivo studies do not show clear effects of PGs in the regulation of obesity. Moreover, PG receptor gene-knock-out mice are not affected like the cells observed in in vitro studies. The explanation of the problems is quite difficult. As PGs have a variety of physiological functions, studies using gene-knock-out mice might not be appropriate to elucidate the functions of PGs in obesity. The precise in vivo functions of PGs especially those of 15d-PGJ_2_ required further clarification. Tissue- (cell-)specific gene-knock-out mice might be a powerful tool to identify the in vivo function of PGs. Understanding of the mechanisms of PG-mediated regulation of adipogenesis may lead to a novel therapeutic strategy for the treatment of obesity.
